# Spring diet and energy intake of whooper swans (*Cygnus cygnus*) at the Yellow River National Wetland in Baotou, China

**DOI:** 10.1371/journal.pone.0264528

**Published:** 2022-02-28

**Authors:** Li Liu, Xiaoguang Liu, Chao Du, Haitao Fang, Jiyun Zhang, Wenjing Li, Litong Cao, Li Gao

**Affiliations:** 1 Faculty of Biological Science and Technology, Baotou Teachers’ College, Baotou, Inner Mongolia, China; 2 Inner Mongolia Forestry Monitoring and Planning Institute, Hohhot, Inner Mongolia, China; University of Veterinary Medicine Vienna: Veterinarmedizinische Universitat Wien, AUSTRIA

## Abstract

The energy supply of food available at stopover sites plays an important role in the life cycle of migratory birds. The Yellow River National Wetland in Baotou, China, is an essential migration station and a source of energy for migratory birds as it is located at an important intersection between East Asian/Australian and Central Asian flyways. From February to may 2020, we measured diet composition and energy content of whooper swans (*Cygnus cygnus*) by fecal micro-tissue analysis to understand their use of the stopover site and inform conservation. The following results were obtained: (1) whooper swans mainly fed on nine species of plants belonging to four families, including corn (*Zea mays*), reeds (*Phragmites australis*), and Suaeda (*Suaeda glauca*), which is related to the availability of local crops and abundance of plants. (2) The energy provided by crops to whooper swans was significantly higher than that of the most abundant plants in wetlands. *Zea mays* was the most consumed crop, and other abundant wetland plants played complementary roles. (3) The daily energy intake of whooper swans was 1393.11 kJ, which was considerably higher than their daily energy consumption. This suggested that the wetlands and the surrounding farmlands provide energy for the whooper swans to continue their migration. In order to protect migratory whooper swans, protection of important refuelling areas such as our study site should be implemented to provide sufficient energy supplies for continuing migration.

## 1 Introduction

Wetlands are important ecological systems that aid in preventing floods and droughts, purifying water quality, and regulating climate [1]. Waterbirds typically depend on wetlands for their abundant food availability and habitat suitability [2]. Abundance and distribution of waterbirds can reflect the structure and functions of wetlands, making them important bio-indicators for wetland health [3]. Wetlands also often act as stopover sites that link the breeding grounds with the wintering grounds for migratory birds. They provide energy resources and resting places for migratory birds, and play an important role in completing their life history [4]. Birds derive energy for migration mainly from the fat stored in the body. Thus, the duration of migration is mainly determined by the time required to replenish energy during rest, i.e., the habitat quality [5, 6]. In recent years, due to the degradation and loss of natural wetlands, food resources have been reduced or have even disappeared. Therefore, waterbirds have been forced to abandon natural wetlands and move to farmland for food [7]. In the long-term development of agricultural practices in China, a large number of wild species have adapted and even become dependent on farmland habitats. However, migratory birds that enter farmland for feeding are posed with new problems, such as human interference, pesticide intake, and limited availability of habitats [7–9]. Therefore, it is crucial to conduct research on the food composition and energy supply of waterbirds in important resting areas to assess whether natural wetlands can still provide sufficient quality food to maintain population migration. In addition, it is vital to study the role of farmland in the energy supply process of migrating birds, and to formulate effective management plans.

The Yellow River National Wetland of Baotou is an important refueling station for migratory birds, because of its location at an important crossroad of the East Asia/Australian and Central Asian flyways [10]. We studied whooper swans (*Cygnus cygnus*), a migratory species that arrives at the wetland to replenish energy in early February every year. The migration peaks in mid-March and gradually reduces from early April. Li et al. (2017) indicated that the Baotou Yellow River National Wetland is an important resting station for migratory birds such as whooper swans. The floodplain formed by the farmland surrounding the wetland provides sufficient food for the swans. Satellite tracking results showed that 66.7% of whooper swans stop in this area for 20–40 days to replenish their energy and then continue to fly to Xinjiang in western China and Mongolia to breed [11, 12]. The energy supply at the stopover site is closely related to the ability of migratory birds to successfully complete the migration process, as well as the successful selection of breeding sites and reproductive success [13]. Energy is derived from the use of different food resources by migratory birds; however, the lack of relevant data hinders the protection of this species. Therefore, in this study, we aimed to elucidate the food composition and energy intake of whooper swan, the representative species of spring migration in Baotou Yellow River National Wetland. The findings of this study will provide a scientific basis for the protection of this species, and play a positive role in promoting quality assessment of the wetland ecological environment and maintenance of biodiversity.

## 2 Materials and methods

### 2.1 Study area

Baotou Yellow River National Wetland (109°25′51′′–111°1′36′′E, 40°14′39′′– 40°33′20′′N) is located on the south of Baotou, Inner Mongolia, and it lies on the north bank of the Yellow River, with a total length of approximately 220 km and a total area of 293.39 km^2^. The wetland has a warm temperate continental monsoon climate, with an average annual temperature of 8.5°C and an average annual precipitation of 307.4 mm. The coldest temperature was approximately −34.4°C in January, and the hottest temperature was 38.4°C in July [14]. The water level of the wetland fluctuates greatly. The freezing period is from late November to mid-February. During this period, the temperature is low, the river is gradually narrowed, and the water level rises. The farmland around the wetland is flooded, and the crops are sealed by ice water. The temperature gradually increases in March, and crops grow to provide abundant food resources for migratory birds. The dominant plants are *Phragmites australis*, *Suaeda glauca*, and *Chenopodium album*. The main crops planted around the wetland are *Zea mays*, *Triticum aestivum*, and *Oryza sativa* [14].

### 2.2 Ethics statement

The current study was performed in accordance with the recommendations on animal care and ethics of China. Noninvasive techniques [15] were used to collect the fecal samples. The Animal Ethics and Welfare committee (AEWC) of Baotou Teachers College approved the implementation of the project. The management authority of Baotou Yellow River National Wetland approved the collection of whooper swan fecal samples.

### 2.3 Sample collection

**Fig 1:**
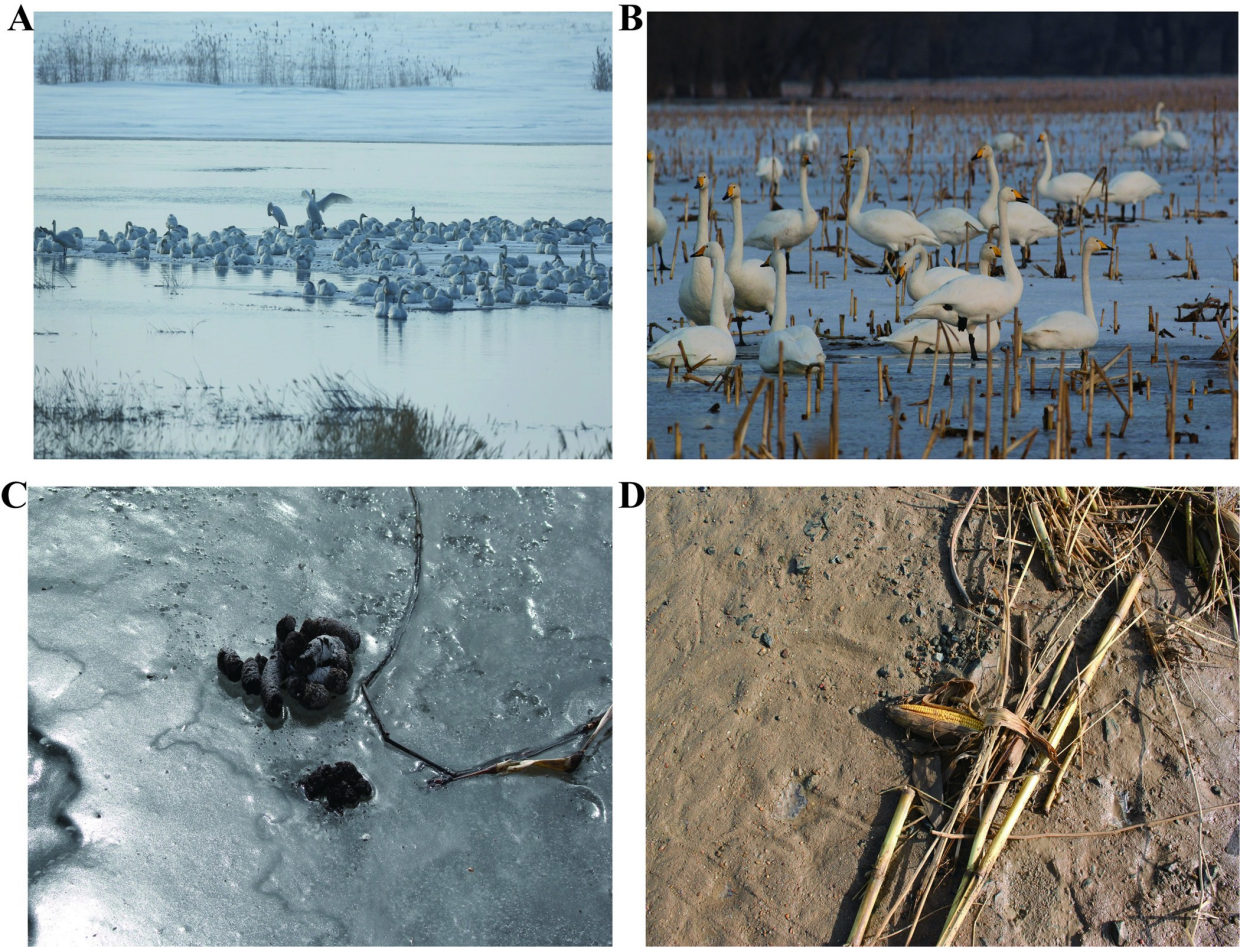
The main whooper swan habitat types and faeces at the study site, the Yellow River National Wetland of Baotou. (A) Whooper swans resting in a natural wetland; (B) Whooper Swan feeding in a field (corn field); (C) The droppings left by Whooper Swans on the ice; (D) The Whooper Swan’s main food is corn.

All fecal samples were collected from the wetland in early February to mid-April 2020. Based on previous studies and the latest waterbird survey, sites with large flocks of whooper swans (i.e., more than 200 individuals) were selected (Fig 1). Whooper swans generally leave feces at the sleeping sites. For the investigation, the resting position was examined on the first night, and its lying position was confirmed again the next morning. Fresh fecal samples were collected after the swans left. The surface detritus of the collected fecal samples was removed and stored individually in paper bags. Disposal gloves were changed for each sample to avoid cross contamination. To avoid repeated sampling and to make sure samples were from different individuals, only samples at a distance of >2 m from each other were collected. In total, 86 fecal samples were collected from 3 sample sites, with more than 20 samples collected from each sampling site. All samples were transported to laboratory and then stored at −20°C until analysis. During the collection of fecal samples, the potential plants around the foraging grounds of the swans were collected, marked, and brought back to the laboratory for identification.

### 2.4 Diet composition analysis

We performed microhistological examination of fecal samples using the method described by Owen (1975) [16]. The epidermal fragments of plants are not easily digested after passing through the digestive tracts of herbivorous birds. Therefore, the feeding habits can be analyzed based on the feces. The plant tissue that was digested and could not be recognized was not considered for the analysis. The samples were dried at 50°C for 48 h, following which 20 samples from each site were randomly selected and ground to powder, eluted with water, mixed thoroughly, and examined with microscope.

The collected plant samples were categorized into seeds, stems and leaves, and a small amount was crushed using a mortar and dried. The drying method was the same as that of the fecal sample. We then compared the fragments in fecal samples with the plant specimens collected from the Yellow River National Wetland. We examined the suspension under a light microscope at 40× magnification for species identification and 10× magnification for quantification statistics.

The composition of the food was quantified by measuring the relative frequency and density of the fragments [17], and *i* represented the proportion of plant species in the food, which can be expressed as:

$$F=1-e^{-d}$$ (1)

where *F* is relative frequency, *e* is the natural logarithm, *d* is the mean fragment density which can be determined by the number of fragments (*n*) and the number of microscope fields examined (*k*) using the following equation:

$$d=\frac{n}{k}$$ (2)

Fragments from m different plant species were randomly distributed in the microscope fields, and the average density of each plant (average number of fragments per field) had no relevance to other species. Relative particle densities (*r*_*i*_), which were estimated by the relative dry weights of each plant in the diet sample, can be calculated by the following equation:

$$r_{i}=\frac{d_{i}}{\sum_{i=1}^{m}d_{i}}$$ (3)

where *i* = l,…, m and *d*_*i*_ is the average density for each species.

### 2.5 Estimation of daily net energy balance

The daily metabolizable energy intake is the energy obtained through food minus the energy excreted in feces. Energy expenditure is the energy lost through metabolism (activity + rest + temperature). The daily net energy balance can be estimated by subtracting the energy expenditure from the metabolizable energy intake.

#### 2.5.1 Energy obtained through food

First, the average daily food intake of the whooper swan was estimated by calculating the energy obtained from food (Eq 4). This value was then calculated based on the proportion of each plant in the food composition and the corresponding energy. Plant energy content was measured by Parr® 6100 Oxygen and Nitrogen Calorimeter (PARR, USA).

$$I_{df}=0.648BW^{0.651}$$ (4)

where *I*_*df*_ is the food consumption rate per day (dry weight, g/d) and *BW* refers to body weight (g). Food consumption rates were estimated by allometric regression models [18]. The weight of whooper swan in this study mainly refers to the weight of the swan described by Kear (2005) [19]; the average *BW* of the whooper swans was 9700 g. By multiplying the value obtained by the proportion of food and energy, the daily energy intake (*DEI*) of whooper swans was calculated using the folwing equation:

$$DEI=\overset{m}{\underset{i=1}{\sum }}I_{df}\cdot r_{i}\cdot Q_{i}$$ (5)

In this equation, *Q*_*i*_ is the energy of the plant per unit mass and *r*_*i*_ is the relative density, i.e., the relative dry weight of each plant in the sample.

#### 2.5.2 Calculation of fecal energy

The energy excreted in the fecal matter was obtained by multiplying the daily total defecation mass (*m*_*T*_) by the caloric values of the dropping per unit mass (*Q*_*d*_) (kJ/g dry weight). The daily total defecation mass was calculated from the mean dropping interval (*t*_*d*_), average dropping mass (*m*_*d*_), and total time spent active (*t*_*A*_). The *m*_*T*_ was calculated using the method described by Wang et al. (2013) [20] using the following equation:

$$m_{T}=\frac{t_{A}}{t_{d}}\cdot m_{d}$$ (6)

where *t*_*A*_ is the number of daily active hours obtained by full-day observations. The number of daily active hours was estimated according to weekly activity time observed. The average dropping interval was assessed by at least 30 direct observations of the interval of two consecutive droppings of randomly selected individuals every week, and averaged as *t*_*d*_.

#### 2.5.3 Calculation of metabolizable energy intake (*MEI*)

The metabolizable energy intake (*MEI*) was calculated as follows:

$$MEI=DEI-Q_{d}\cdot m_{T}$$ (7)

#### 2.5.4 Estimating daily energy expenditure

Daily energy expenditure was estimated by combining the energy cost of different behaviors based on basal metabolic rate (*BMR*, kJ/h), calculated according to the methods described by Reynolds and Lee (1996) [21] as:

$$BMR=0.176\times BW^{0.635}$$ (8)

The energy expenditure for flying, feeding, alertness, resting (sleeping, standing, sitting, and floating combined), and other activities (swimming, preening, drinking, and among others) were 14 × *BMR*, 2 × *BMR*, 2.1 × *BMR*, 1.3 × *BMR*, and 2.3 × *BMR*, respectively [22]. These values were almost the same as those for the larger bean geese (*Anser fabalis serrirostris*), greater white-fronted geese (*Anser albifrons*), and lesser white-fronted geese (*Anser erythropus*), which were studied by other methods [23].

Low temperatures increased the energy expenditure. Whooper swans must increase their heat production to maintain body temperature below the ‘lower critical temperature’ (*LCT*, °C). *LCT* was calculated by the equation used by Calder and King (1974) [24]:

$$LCT=T_{0}-4.73\times BW^{0.274}$$ (9)

where body temperature (T_0_) was assumed to be 40°C. The daily heat loss below *LCT* (*H*_*LCT*_, kJ/day) was calculated following the method of Lefebvre and Raveling (1967) [25]:

$$H_{LCT}=a\times (T_{b}-LCT)\times H_{b}$$ (10)

The coefficient ‘a’ (3.05) was estimated by a quadratic regression of heat loss coefficient against body mass, based on the data provided by Lefebvre and Raveling (1967) [25]. *T*_*b*_ and *H*_*b*_ are the average temperature and number of hours below *LCT*, respectively. We used the daily maximum and minimum temperatures of Baotou city, located approximately 30 km away from the study area.

### 2.6 Data analysis and statistics

In order to clarify the level of energy provided by different plants to the whooper swan in the food composition, the square root of arcsine conversion was carried out on the energy intake of different plants by the whooper swan and subjected to principal components analysis (PCA) in SPSS v. 20.0. The PCA results were plotted to determine the energy contribution of different plants to whooper swan. The difference in energy supply between crops and dominant plants to whooper swans was analyzed by a non-parametric Kruskal-Wallis test (*P* = 0.05) (SPSS). The differences in feeding frequencies of different plants were analyzed by one-way analysis of variance (ANOVA) to clarify the differences in energy supply between crops and wetland plants for whooper swan. The crops in this study are defined as: cultivated plants in the farmland, such as corn, rice, wheat, and other crops. Wetland plants are defined as: non-divisional cultivation of plants that grow naturally in wetlands, such as *Phragmites australis*, *Suaeda salsa*, *Artemisia sphaerocephala*, and other species.

## 3 Results

### 3.1 Food composition

The results of the diet analysis showed that (Table 1) the whooper swans fed mainly on nine species of plants from four families at the Yellow River National Wetland in Baotou. *Zea mays* was the most abundant component of the diet, comprising 50.27% of the overall diet, followed by *P*. *australis* (29.49%), *S*. *glauca* (15.05%), *O*. *sativa* (1.91%), *Artemisia sieversiana* (1.40%), *C*. *album* (0.83%), *Chenopodium hybridum* (0.48%), *Polygonum lapathifolium* (0.16%), and *Ixeris denticulata* (0.08%). Further analysis showed that the whooper swans fed on crops with significantly higher frequencies than on the most abundant wetland plants (*P* = 0.01, *d*_*f*_ = 59). In terms of the feeding frequencies of swans with respect to the different plant families available, significant differences were detected among Gramineae, Chenopodiaceae, Compositae, and Polygonaceae (*P* = 0.00, *d*_*f*_ = 59). However, there was no significant difference between Compositae and Polygonaceae as the whooper swans fed mainly on gramineous plants.

**Table 1:** Food composition of whooper swan.

Family	Plant species	Feeding organ	Relative frequency (%)	Relative density (%)
Gramineae	*Zea mays*	Seed	30.00	50.27
		Leaf	13.58	
	*Phragmites australis*	Leaf	22.62	29.49
		Seed	2.31	
		Stem	1.49	
	*Oryza sativa*	Seed	1.82	1.91
		Leaf	0.07	
Chenopodiaceae	*Suaeda glauca*	Leaf	7.26	15.05
		Stem	7.23	
	*Chenopodium album*	Seed	0.83	0.83
	*Chenopodium hybridum*	Leaf	0.48	0.48
Compositae	*Ixeris denticulata*	Leaf	0.08	0.08
	*Artemisia sieversiana*	Leaf	1.03	1.40
polygonaceae	*Polygonum lapathifolium*	Stem	0.16	0.16
	others		0.32	0.32

### 3.2 Analysis of plant energy contribution

To clarify the difference in energy provided by various plants to the whooper swan diet, PCA analysis was performed on the energy values of the nine most abundant plants in the droppings (Table 2). The first two PCA axes accounted for 46.26% (25.53 and 20.73%, respectively) of the energy contribution. The first axis was characterized by high positive loadings of *Z*. *mays* and *I*. *denticulata*, and high negative loadings of *P*. *australis*. The second axis reflected the positive influence of *C*. *album*, *A*. *sieversiana*, and *O*. *sativa*.

**Table 2:** The energy loadings of the different plant constituents.

Plant species	Eigenvector axis 1	Eigenvector axis 2
*Zea mays*	**0.822**	-0.005
*Phragmites australis*	**-0.782**	-0.054
*Chenopodium hybridum*	0.550	-0.343
*Ixeris denticulata*	**0.558**	-0.332
*Oryza sativa*	0.080	**0.533**
*Chenopodium album*	0.011	**0.842**
*Artemisia sieversiana*	-0.015	**0.796**
*Polygonum lapathifolium*	-0.349	-0.125
*Suaeda glauca*	-0.457	-0.229

Note: Table 2 Lists the major plant species identified in the fecal sample and its corresponding energy principal component analysis results. The values in table represents the eigenvectors for each species, emboldened values indicated the three plant groups making the most contribution to each axis.

**Fig 2:**
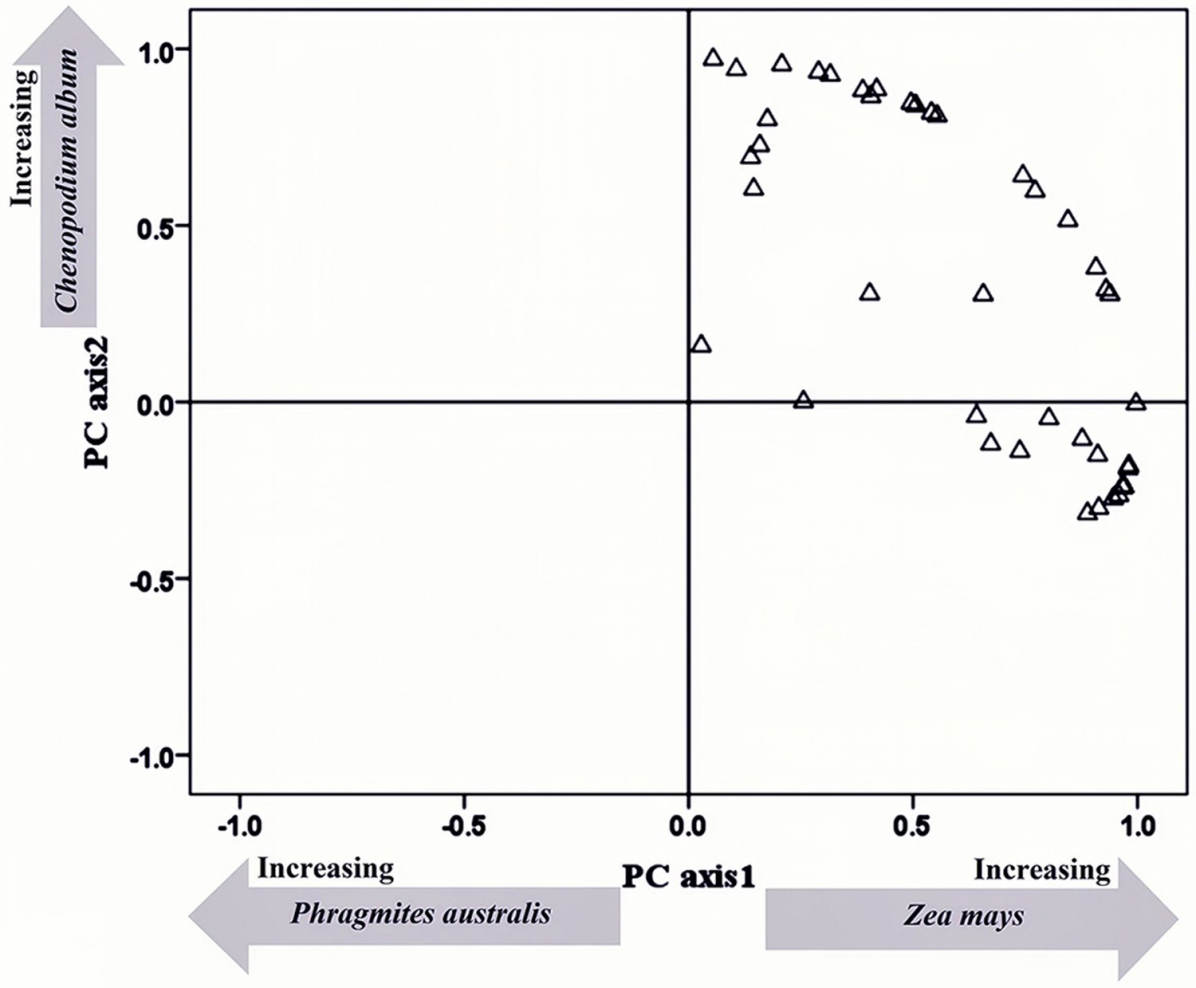
Principal components analysis ordination of the content of collections of fecal.

**Fig 3:**
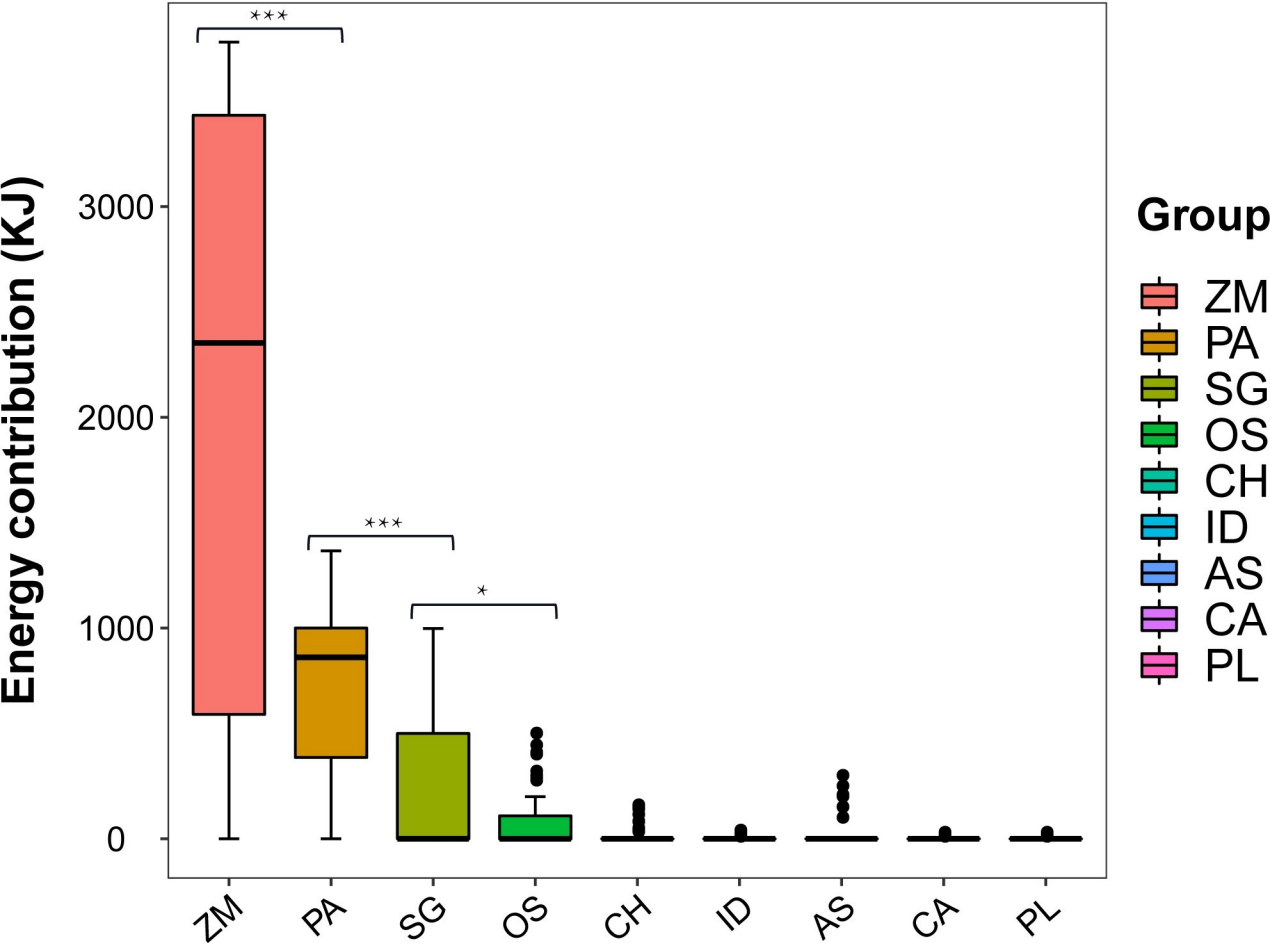
The energy contribution of different plants to whooper swan. The energy contribution of corn (*Zea mays*) to whooper swan was significantly higher than other plants (*p*<0.05).*p<0.*05*, **p<0.01, ***p<0.001. ZM:*Zea mays*; PA:*Phragmites australis*; SG:*Suaeda glauca*; OS:*Oryza sativa*; CH:*Chenopodium hybridum*; ID:*Ixeris denticulata*; AS:*Artemisia sieversiana*; CA:*Chenopodium album*; PL:*Polygonum lapathifolium*.

The distribution map of the energy contribution of all samples is drawn with the two principal components as the coordinate axes (Fig 2). Results showed that the main energy provided for whooper swan migration were crops and wetland dominant plants, and the distribution of energy supply among samples is concentrated. In order to clarify the difference in energy provided by different plants, a non-parametric test was carried out on the energy data of all plants in food composition (Fig 3). The results showed that the energy provided by corn to whooper swans was significantly higher than other dominant plants in wetland (p <0.01) and it plays a leading role in the energy supply of whooper swan.

### 3.3 Daily metabolizable energy intake and consumption

Most of the plants that the whooper swans fed on were corn seeds, reed leaves, and corn leaves, and their respective metabolizable energy intakes were: 1458.61 kJ d^−1^, 796.13 kJ d^−1^, and 415.69 kJ d^−1^ (Table 3). The daily metabolizable energy intake (*MEI*) of whooper swans was calculated to be 2682.64 kJ. This energy value supports a variety of behavioral activities, including flying, feeding, alertness, and resting. The energy consumption was estimated to be 1289.53 kJ. Flying accounted for 50.49% of the total daily energy consumption (*DEE* tot) of whooper swans, while feeding, alertness, and resting accounted for 29.76%, 0.82%, and 14.40%, respectively. In addition, the energy excreted based on the droppings was 605.68 kJ. The difference between daily metabolizable energy intake and daily energy consumption is the energy budget of the whooper swan; this was estimated as 1393.11 kJ.

**Table 3:** The metabolizable energy intake (*MEI*) of whooper swan to different plants.

Plant species	Feeding organs	Total energy (kJ/g)	ADF (%	The daily energy intake (kJ/d)
*Zea mays*	Seed	16.68	3.98	**1458.61**
	Leaf	17.22	35.18	**415.69**
*Phragmites australis*	Leaf	18.84	35.45	**796.13**
	Seed	20.27	39.38	64.81
	Stem	17.47	45.35	36.55
*Suaeda glauca*	Leaf	13.29	25.90	189.51
	Stem	14.73	37.19	177.30
*Oryza sativa*	Seed	16.43	15.51	65.19
	Leaf	15.10	43.50	1.52
*Artemisia sieversiana*	Leaf	16.90	26.69	44.26
*Chenopodium album*	Seed	17.62	27.23	21.71
*Chenopodium hybridum*	Leaf	13.76	29.04	11.96
*Polygonum lapathifolium*	Stem	15.38	56.47	2.74
*Ixeris denticulata*	Leaf	17.99	36.08	2.35

## 4 Discussion

### 4.1 Food composition diversity

According to current research, whooper swans have diverse diets that varies spatially and temporally. By studying wintering home range and habitat, Jia et al. (2019) found that whooper swans mainly feed on reeds and cattails during the early stage of overwintering in Sanmenxia Wetland, China [26]. Based on a study on trace element exposure, Wang et al. (2017) found that whooper swans mainly feed on eelgrass, macroalgae, and *Zostera marina* when wintering in a marine lagoon in northern China [27]. By studying seasonal variation in energy gain and resource patterns, Wood et al. (2019) found that whooper swans shift from using predominantly root crops (e.g., sugar beet and potatoes) in early winter to using mostly cereals (e.g., wheat) in late winter [28]. In the present study, we analyzed the feeding habits of whooper swans in the Baotou Yellow River National Wetland, and found that they mainly fed on nine species of plants from four families. These plants were a combination of local crops and abundant wetland plants with *Z*. *mays*, *P*. *australis*, and *S*. *glauca* being the most important to the whooper swan diet in this location. The diverse diet of whooper swans may be an adaptation to seasonal fluctuations in food resources and an attempt to optimize energy accumulation.

### 4.2 Energy contribution of different plants

According to the results of the current study, migratory birds can gain more energy from shifting their habitat from wetlands to farmlands. Consistent with our findings, Fox et al. (2017), who studied the feeding habitat of wild geese, found that farmlands provide considerably more food resources for geese than the traditional wintering grounds of natural wetlands, especially by providing corn seeds that contain high protein and fat contents, and can provide a great deal of energy for the geese [29]. Yu et al. (2017) reported that the shift from natural habitat to farmland for geese can greatly increase the rate of food intake, so that the geese can obtain a better physical condition throughout the annual cycle, increase reproductive output, and expand the carrying capacity of non-breeding areas [7]. Additionally, satellite tracking results also confirmed that the main habitat of whooper swans in the spring migration season is farmland. Therefore, the present study concluded that feeding maize in Baotou Yellow River National Wetland could provide energy guarantee for the continued migration of whooper swans. Among the food components, the crop corn had the highest relative density, which accounted for more than 50% of the food components. Energy supply analysis showed that the energy load factor contributed by corn was higher than that of other plants. This indicated that *Z*. *mays* plays a leading role in the energy supply of whooper swan migration.

In addition, the energy contribution analysis results showed that the dominant plants (*Phragmites australis*, *Ixeris denticulata*, *Chenopodium album*, and *Artemisia sieversiana*) also provide an important energy supplement in the wetland for the whooper swan although the feeding frequency is relatively low in comparison to *Z*. *mays* resulting in being considered as an overall limited energy source. We found that large areas of native wetlands were replaced by farmland [30], and the habitat quality of existing native wetlands has deteriorated leading to a reduction of the dominant plants in the wetland. This may also explain why the abundant wetland plants provide less energy than crops.

### 4.3 Energy accumulation

During migration, birds face various adverse factors such as different habitat types, climate conditions, and natural enemies [31]. They need a quick energy replenishment to reduce the stress they face during migration [32]. Foraging theory predicts that animals select patches that offer the highest net rate of energy gain [33, 34]. Foraging on higher biomass patches can increase foraging success and increase energy intake rate [35]. Anatidae species often use a strategy of energy maximization. Chen et al. (2019) reported that the daily energy intake of barnacle geese (*Branta leucopsis*) was maximized by digging tubers of intermediate depth (11–20 cm) [36]. At the end of overwintering, the daily energy intake was 2624 kJ, which was higher than *DEE*, in order to accumulate body mass and reserve energy for spring migration. Prop et al. (2004) found that barnacle geese (*Branta leucopsis*) accumulated the largest amounts of energy reserves when staging in the agricultural habitat, to make up for the energy expended during migration [37]. The present study showed that the highest feeding frequency and energy supply of whooper swans were for corn crop that had been flooded by the Yellow River in a relatively large area, indicating that whooper swans fed on relatively more quantity and easily available food to obtain the maximum energy supply during the limited time available for stopover. The daily energy intake was 2682.64 kJ, which was higher than the total energy consumption (*DEE* tot) of 1289.53 kJ. Consistent with the findings of other studies, energy accumulation was observed, which can provide the energy to continue the migration.

Liang et al. (2020) indicated that hydrology-climate changes and food availability could be the primary drivers that result in a loss of waterbird diversity [38, 39]. Therefore, in the present study, replenishing of energy by whooper swans is not only related to the flooding of unharvested farmland, but also affected by the local climate. The area is located at a higher latitude in the Yellow River Basin, with an altitude of approximately 1000 m above sea level and the temperature changes drastically. The freezing period is from late November to mid-February. The average daily temperature is below −10°C. The decrease in temperature makes the river channel narrow and the water level rises, thereby flooding the surrounding farmland. In late February, the weather becomes warmer, and submerged crops surface, providing large amounts of food for migratory birds, which is one of the reasons why the whooper swans replenish their energy here.

In summary, the Baotou Yellow River National Wetland is an important resting and an refueling stopover site for many migratory birds represented by swans. The crop corn plays a major role in energy accumulation during the migration of whooper swans, and other wetland plants play an important supplementary role. Therefore, in order to protect the migratory birds such as whooper swans, measures should be implemented to protect the wetland and surrounding farmland to provide suitable conditions for energy supply for migratory birds.

## Supporting information

S1 Table The analysis results of sample 1–20. (XLSX)

S2 Table The analysis results of sample 21–40. (XLSX)

S3 Table The analysis results of sample 41–60. (XLSX)

S4 Table The different tissues of wetland plants correspond to the plants in the fecal sample. (XLSX)

S5 Table Sampling information. (XLS)

S6 Table The Wilcoxon test results of the energy supply of different plants to whooper swan. (DOCX)

## References

[1] Brander LM, Florax RJ, Vermaat JE. The empirics of wetland valuation. A comprehensive summary and a meta-analysis of the literature. Environ Resour. 2006; 33:223–250. doi: 10.1007/s10640-005-3104-4

[2] Fan Y, Zhou L, Cheng L, Song Y, Xu W. Foraging behavior of the Greater White-fronted Goose (*Anser albifrons*) wintering at Shengjin Lake: diet shifts and habitat use. Avian Ses. 2020; 11(1):65–73. doi: 10.1186/s40657-020-0189-y

[3] Fox AD, Cao L, Zhang Y, Barter M, Zhao M, Meng F, et al. Declines in the tuber-feeding waterbird guild at Shengjin Lake National Nature Reserve, China–abarometer of submerged macrophyte collapse. Aquatic Conservation: Aquat. Conserv. 2011;21:82–91. doi: 10.1002/aqc.1154

[4] Cao L, Meng FJ, Zhao QS. Understanding Effects of Large-scale Development on Bird Migration and Habitats Through Cutting Edge Avian Monitoring Techniques. Bulletin of the Chinese Academy of Sciences. 2021;36(4): 436–447. doi: 10.16418/j.issn.1000-3045.20210309002

[5] Wang X, Fox AD, Cong P, Cao L. Food constraints explain the restricted distribution of wintering Lesser white-fronted Geese Anser erythropus in China. Ibis. 2013; 155: 576–592. doi: 10.1111/ibi.12039

[6] Alerstam T, Lindstrom A. Optimal Bird Migration: The Relative Importance of Time, Energy, and Safety. Bird Migration. 1990;331–351. doi: 10.1007/978-3-642-74542-3_22

[7] Yu H, Wang X, Cao L, Zhang L, Jia Q, Lee H, et al. Are declining populations of wild geese in China ’prisoners’ of their natural habitats? Curr Biol. 2017; 27(10): R376–R377. doi: 10.1016/j.cub.2017.04.037 28535385

[8] Li L, Hu R, Huang JK, Bürgi M, Zhu ZY, Zhong J, et al. A farmland biodiversity strategy is needed for China. Nat Ecol Evol. 2020;4:772–774.doi: 10.1038/s41559-020-1161-2 32221478

[9] Liang J, Gao X, Zeng G, Hua S, Zhong M, Li X, et al. Coupling Modern Portfolio Theory and Marxan enhances the efficiency of Lesser White-fronted Goose’s (Anser erythropus) habitat conservation. Sci Rep. 2018;8(1):214. doi: 10.1038/s41598-017-18594-2 29317759 PMC5760730

[10] Liu L, Liu XG, Sun Y, Pu ZH, Xu HY, Li WX, et al. Trace Elements in the Feathers of Waterfowl from Nanhaizi Wetland, Baotou, China. Bull Environ Contam Toxicol. 2019;102(6):778–783. doi: 10.1007/s00128-019-02596-z 30918995

[11] Li SH, Meng WY, Chen LX, Li YF, Gao RY, Ru WD. The spring waterbird community and home range of the whooper swan Cygnus cygnus at the upper and middle reaches of Yellow River in Inner Mongolia, China. Chinese Journal of Ecology. 2017; 36: 1910–1916. doi: 10.13292/j.1000-4890.201707.035

[12] Li YB, Li MH, Li YL, Tian JM, Bai XL, Yang C, et al. Outbreaks of Highly Pathogenic Avian Influenza (H5N6) Virus Subclade 2.3.4.4h in Swans, Xinjiang, Western China, 2020. Emerg Infect Dis. 2020; 26(12): 2956–2960. doi: 10.3201/eid2612.201201 33030424 PMC7706961

[13] Kölzsch A, Müskens GJDM, Kruckenberg H, Glazov P, Weinzierl R, Nolet BA, et al. Towards a new understanding of migration timing: slower spring than autumn migration in geese reflects different decision rules for stopover use and departure. Oikos. 2016; 125(10): 1496–1507. doi: 10.1111/oik.03121

[14] Liu L, Du C, Sun Y, Liu J, Pu Z, Liu X. Trace element distribution in tissues and risk of exposure of ruddy shelduck wintering in Nanhaizi Wetland, Baotou, China. Environ Sci Pollut Res Int. 2020;27(6):6429–6437. doi: 10.1007/s11356-019-07132-4 31873889

[15] Darimont CT, Reimchen TE, Bryan HM. Faecal‐centric approaches to wildlife ecology and conservation; methods, data and ethics. Wildlife Biology in Practice. 2008; 4(2):14. doi: 10.2461/wbp.2008.4.7

[16] Owen M. An assessment of fecal analysis technique in waterfowl feeding studies. J Wildl Manage. 1975; 39: 271–279. doi: 10.2307/3799903

[17] Johnson MK, Wofford H, Pearson HA. Digestion and fragmentation: influence on herbivore diet analysis. J Wildlife Manage.1983; 47: 877–879.

[18] Nagy KA. Field metabolic rate and food requirement scaling in mammals and birds. Ecol Monogr. 1987;57:111–128. doi: 10.2307/1942620

[19] Kear J. Ducks, Geese, and Swans. Oxford University Press, 2005.

[20] Wang X, Zhang Y, Zhao M, Cao L, Fox AD. The benefits of being big: effects of body size on energy budgets of three wintering goose species grazing Carex beds in the Yangtze River floodplain. China J Orn. 2013;154: 1095–1103. doi: 10.1007/s10336-013-0979-7

[21] Reynolds PS, Lee RM. Phylogenetic analysis of avian energetics: passerines and nonpasserines do not differ. Am Nat. 1996;147: 735–759. doi: 10.2307/2463088

[22] King JR. Seasonal allocation of time and energy resources in birds. Avian energetics.1974; 4–85.

[23] Clausen KK, Clausen P, Fox AD, Fælled CC, Madsen J. Varying energetic costs of Brent Geese along a continuum from aquatic to agricultural habitats: the importance of habitat-specific energy expenditure. J Ornithol. 2013; 154: 155–162. doi: 10.1007/s10336-012-0881-8

[24] Calder WA. Thermal and caloric relations of birds. Avian biol.1974; 4: 259–413.

[25] Lefebvre EA, Raveling DG. Distribution of Canada Geese in winter as related to heat loss at varying environmental temperatures. J Wildl Manage. 1967;31: 538–546.

[26] Jia R, Li SH, Meng WY, Gao RY, Ru WD, Li YF, et al. Wintering home range and habitat use of the whooper swans (Cygnus cygnus) in Sanmenxia Wetland, China. Ecol Res. 2019; 34:637–643. doi: 10.1111/1440-1703.12031

[27] Wang F, Xu SC, Zhou Y, Wang PM, Zhang XM. Trace element exposure of whooper swans (Cygnus cygnus) wintering in a marine lagoon (Swan Lake), northern China. Mar Pollut Bull. 2017;119: 60–67. doi: 10.1016/j.marpolbul.2017.03.063 28392089

[28] Wood KA, Hilton GM, Newth JL, Rees EC. Seasonal variation in energy gain explains patterns of resource use by avian herbivores in an agricultural landscape: Insights from a mechanistic model. Ecol Model. 2019; 409:108762. doi: 10.1016/j.ecolmodel.2019.108762

[29] Fox AD, Abraham KF. Why geese benefit from the transition from natural vegetation to agriculture. Ambio. 2017; 46: 188–197. doi: 10.1007/s13280-016-0879-1 28215009 PMC5316322

[30] Wang Y, Yu LH, Li WP, Gao JT, Liu JL. Ecological Health Assessment in Different Areas of Baotou Section of the Yellow River Wetlands Based on PSR Model. Journal of Inner Mongolia University (Natural Science Edition). 2016; 47: 328–336.

[31] Wang X, Cao L, Fox AD, Fuller R, Griffin L, Mitchell C, et al. Stochastic simulations reveal few green wave surfing populations among spring migrating herbivorous waterfowl. Nat Commun. 2019;10(1):2187. doi: 10.1038/s41467-019-09971-8 31097711 PMC6522631

[32] Thomas KL, Adriaan MD, Henk PJ, Willem B, Jasper K, Stefan HHS, et al. Nocturnal foraging lifts time constraints in winter for migratory geese but hardly speeds up fueling. Behav Ecol. 2021;.32: 539–552. doi: 10.1093/beheco/araa152 34104110 PMC8177807

[33] Emlen JM. The role of time and energy in food preference. Am Nat. 1966;100: 611–7.

[34] Pyke GH. Optimal foraging theory: a critical review. Annu Rev Ecol Syst. 1984; 15: 523–75. doi: 10.1146/annurev.ecolsys.15.1.523

[35] Bergman CM., Fryxell JM, Gates CC, Fortin D. Ungulate foraging strategies: energy maximizing or time minimizing? J Anim Ecol. 2001;70: 289–300. doi: 10.2307/2693426

[36] Chen Y, Zhang Y, Cao L, de Boer WF, Fox AD. Wintering Swan Geese maximize energy intake through substrate foraging depth when feeding on buried Vallisneria natans tubers. Avian Res. 2019; 10: 163–170. doi: 10.1186/s40657-019-0145-x

[37] Prop J, Black JM. Food intake, body reserves and reproductive success of bar nacle geese Branta leucopsis staging in difffferent habitats. Norsk Polarinst. Skrift. 1998; 200: 175–194. doi: 10.1139/cjb-79-3-293

[38] Liang J, Meng Q, Li X, Yuan Y, Peng Y, Li X, et al. The influence of hydrological variables, climatic variables and food availability on Anatidae in interconnected river-lake systems, the middle and lower reaches of the Yangtze River floodplain. Sci Total Environ. 2021;768:144534. doi: 10.1016/j.scitotenv.2020.144534 33454478

[39] Liang J, Peng Y, Zhu Z, Li X, Yuan Y. Impacts of changing climate on the distribution of migratory birds in China: Habitat change and population centroid shift. Ecological Indicators, 2021, 127(1764):107729. doi: 10.1016/j.ecolind.2021.107729

