# Supplementary material for: Spring diet and energy intake of whooper swans (Cygnus cygnus) at the Yellow River National Wetland in Baotou, China
Source: PLoS One. 2022 Feb 28;17(2):e0264528. doi: 10.1371/journal.pone.0264528 (PMC8884505; doi:10.1371/journal.pone.0264528)
Supplement: S6 Table — (DOCX) [file pone.0264528.s006.docx]

**S6 Table. The Wilcoxon test results of the energy supply of different plants to whooper swan**

|  | ZM | PA | SG | OS | CH | ID | AS | CA |
| --- | --- | --- | --- | --- | --- | --- | --- | --- |
| PA | 0.00^***^ |  |  |  |  |  |  |  |
| SG | 0.00^***^ | 0.00^***^ |  |  |  |  |  |  |
| OS | 0.00^***^ | 0.00^***^ | 0.03^*^ |  |  |  |  |  |
| CH | 0.00^***^ | 0.00^***^ | 0.00^***^ | 0.02^*^ |  |  |  |  |
| ID | 0.00^***^ | 0.00^***^ | 0.00^***^ | 0.00^***^ | 0.41 |  |  |  |
| AS | 0.00^***^ | 0.00^***^ | 0.00^***^ | 0.10 | 0.40 | 0.18 |  |  |
| CA | 0.00^***^ | 0.00^***^ | 0.00^***^ | 0.00^***^ | 0.11 | 0.38 | 0.04^*^ |  |
| PL | 0.00^***^ | 0.00^***^ | 0.00^***^ | 0.01^**^ | 0.54 | 0.76 | 0.26 | 0.23 |

ZM:*Zea mays*; PA:*Phragmites australis*; SG:*Suaeda glauca*; OS:*Oryza sativa*; CH:*Chenopodium hybridum*; ID:*Ixeris denticulata*; AS:*Artemisia sieversiana*; CA:*Chenopodium album*; PL:*Polygonum lapathifolium.*

**p<0.05*, ***p*<0.01, ****p*<0.001.
